# A rare case of primary thyroid amyloidosis

**DOI:** 10.1016/j.ijscr.2018.10.033

**Published:** 2018-10-25

**Authors:** M.A. Cannizzaro, S. Lo Bianco, W. Saliba, S. D’Errico, F. Pennetti Pennella, G. Buttafuoco, D. Provenzano, G. Magro

**Affiliations:** a Department of Medical and Surgical Sciences and Advanced Technologies, G.F. Ingrassia, Catania, Italy; b Resident in General Surgery Training Program, Department General surgery and medical-surgical specialties, Endocrinesurgery Unit, “Policlinico-Vittorio Emanuele” Hospital, Catania, Italy

**Keywords:** Goiter, Amyloidosis, Thyroid, Primary amyloidosis, Thyroid disease

## Abstract

**Introduction:**

Amyloid goiter is due to the deposition of amyloid in the thyroid, resulting with enlargement of the gland and compressive symptoms.

**Case:**

We herein present a case of a 45-year-old male patient who complained of a big swelling in the neck. Ultrasound showed an enlarged thyroid gland with mediastinal involvement. The multinodular appearance was consistent with the diagnosis of multinodular goiter. He had a history of multiple myeloma but no sign of systemic amyloidosis.

**Discussion:**

Thyroid gland was removed and the histopathological examination revealed a diffuse deposition of amyloid associated with metaplastic lipomatosis of the stroma.

**Conclusions:**

The treatment of choice in patients with amyloid goiter is total thyroidectomy to solve compression symptoms.

## Highlights

• The patient complained of dysphagia and respiratory difficulty.
• He had not shown any signs of systemic amyloidosis.
• The patient underwent total thyroidectomy.
• Based on morphological and histochemical features, the diagnosis of “amyloid goiter” was rendered.

## 1 Introduction

Amyloid goiter is due to the deposition of amyloid protein within the thyroid gland, accompanied by fat deposition. The 15% of deposition of amyloid occurs in primary amyloidosis, 20% in the secondary [1]. The deposition of amyloid causes enlargement of the thyroid gland, with compressive symptoms [2]. Therapy is surgical, especially if compressive symptoms are present.

We here in present a rare case of primary thyroid amyloidosis diagnosed on histopathological examination after surgery. Informed consent was received from the patient. The work has been reported in line with the SCARE criteria [3].

## 2 Case report

A 45-year-old male patient presented to our institute in December 2017. He suffered of kidney failure and multiple myeloma for about 10 years. The patient complained of dysphagia and respiratory difficulty. Clinical examination showed a big swelling of the neck. He had not shown any signs of systemic amyloidosis. There were no symptoms suggestive of hypo- or hyper-thyroidism. Ultrasound showed an increased volume of the thyroid gland (right lobe 49 × 38 × 100 mm; left lobe 41 × 34 × 51 mm.) with involvement of the mediastinum. No lateral cervical lymphadenopathy was appreciated. CT and MRI showed diffuse and multinodular enlargement of both lobes of the thyroid gland, no lateral cervical lymphadenopathy (right lobe reaches C2; left lobe reaches the brachio-cephalic trunk). Fine needle aspiration (FNA), performed in one nodule of 2 cm in its greatest dimension, showed the presence of colloid and histiocytes. The patient underwent total thyroidectomy. The post-operative course was unremarkable.

**Fig. 1:**
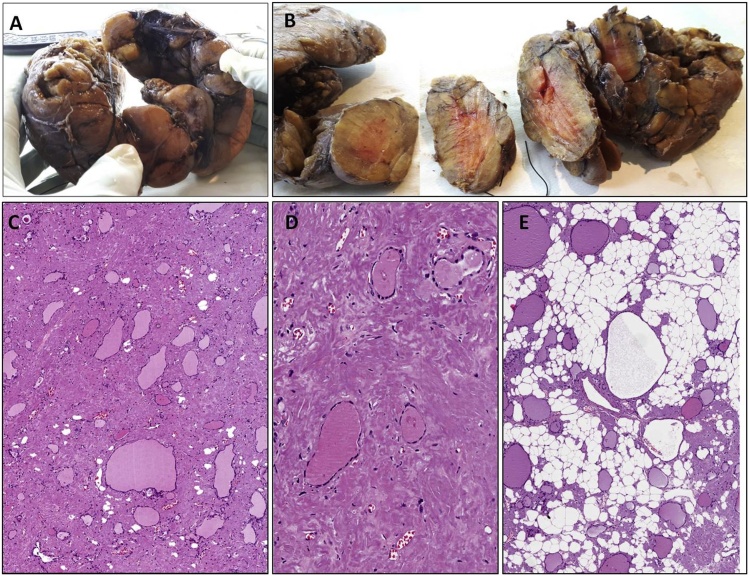
A. Stroma of the thyroid is largely replaced by amorphous eosinophilic material. Residual follicles, some of which with cystic changes, can be seen.

Grossly, thyroid was diffusely enlarged with a nodular external surface (Fig. 1A). The cut surface showed a soft, irregularly nodular and salmon in color parenchyma (Fig. 1B). Histologically there was a diffuse stromal deposition of amorphus eosinophilic material, reminiscent of fibro-sclerotic changes (Fig. 1C and D). Residual normal-sized or cistically dilated thyroid follicles were seen (Fig. 1C and D). Notably some areas showed a variably fatty stromal component characterized by mature adipocytes (Fig. 1E). This component was interpreted as a fatty stromal metaplasia. PAS staining was negative or only weakly positive in the amorphus eosinophilic stromal material. Conversely, a positive staining was obtained with Rosso Congo stain (apple-green birefringence under polarized light). Based on morphological and histochemical features, the diagnosis of “*amyloid goiter*” was rendered.

## 3 Discussion

Amyloidosis is defined as a heterogeneous group of diseases, caused by the alteration of protein folding processes that lead to the formation of insoluble proteins, which are deposited within the tissues, more exactly within the extracellular space. 30 different types of amyloidosis are listed [4]. The fibrillar deposition of amyloid can occur in any tissue. Increased deposition of fibrillar amyloid within tissues compromises organ function. Histologically, amyloid appears as an eosinophilic amorphic material mimicking fibrous tissue. The diagnosis depends on the demonstration of apple-green birifrangennce under polarised light when stained with Congo red.

The classification of amyloidosis is based on the fibril accumulated in the tissue and its origin.

We can distinguish the following main forms: primary, secondary and hereditary forms.

The primary has not known cause and it is often associated with the deposition of AL amyloid fibres. The amyloid proteins, that build up in the tissues, in this condition are known as light chains. They can be either kappa or lambda light chains. AL amyloidosis is caused by a disorder of the plasma cells. Plasma cells, a type of white blood cell are responsible for the production of immunoglobulins or antibodies, which are a type of protein that fights infection. In AL amyloidosis, the light chain proteins are misshapen and produced in excess. They deposit in tissues and can damage one or more organs. Heart, kidneys, nerves, and gastrointestinal system are the most common organs affected. Because AL amyloidosis is associated with the overproduction of plasma cell proteins, it is usually linked to multiple myeloma [5,6].

The secondary amyloidosis is caused by other diseases, such as some type of cancer, often associated to AA deposition. In this class, the amyloid protein, that lodge in the tissues, is called the A protein. AA amyloidosis is associated with chronic diseases, such as diabetes, tuberculosis, rheumatoid arthritis, and inflammatory bowel disease. It may also be linked to aging. AA amyloidosis can affect the spleen, liver, kidneys, adrenal glands, and lymph nodes. Lymph nodes are tiny, bean-shaped organs that fight infection [[7], [8], [9], [10], [11], [12], [13]].

Hereditary amyloidosis is rare. It can be passed from generation to generation within a family. The proteins produced in hereditary amyloidosis may cause problems with the heart and may cause carpal tunnel syndrome and eye abnormalities. The most common subtypes involve a protein called transthyretin (TTR).

Amyloid goitre is defined as the deposition of amyloid in the glandular parenchyma, such as to determine a volumetric enlargement, clinically evident, and atrophy of thyroid follicles [[14], [15], [16]].

Thyroid nodules and Thyroid enlargement are common in the general population. Causes of goiter are: Hashimoto thyroiditis, Graves’s disease, De Quervain and Riedle thyroiditis, thyroid cyst. The most important issue is to exclude malignancy, like papillary thyroid cancer, follicular thyroid cancer, medullary thyroid cancer, thyroid lymphoma and anaplastic thyroid cancer. Diagnosis must be made by histologic test of the thyroid gland. The presence of amyloid goiter must raise the differential diagnosis of medullary carcinoma of the thyroid, multiple myeloma, rheumatoid arthritis, hyalinizing trabecular adenoma, solitary plasmacytoma, infections and familial Mediterranean fever.

This is a rare clinical condition that may be associated with the deposition of adipose tissue within the gland. From a clinical point of view, patients present with a clinically evident, painless and rapidly growing crop (months or years) that can cause progressive airway obstruction, dysphagia and dysphonia [17]. Thyroid deposition of amyloid is very frequent, estimated between 20 and 50% of primary amyloidosis and up to 80% in secondary amyloidosis [18]. The origin of the adipose tissue is unclear. It has been suggested that it may arise from metaplasia of stromal fibroblasts in response to ischemia due to the destruction of capillaries by amyloid deposition [19]. Patients can be clinically hypothyroid, hyperthyroid or euthyroid [[20], [21], [22]].

The imaging features of amyloid goiter may vary depending on the amount of fat and amyloid deposited within the thyroid parenchyma. In cases with amyloid deposition, complex or hypoechoic masses are shown on sonography. Proteinaceous substances within the nodule can cause high intensity on T1-weightes images, and fibrillar amyloid structures can lead to increased intensity on T2-weighted images [23]. On MRI, fatty infiltration causes increased signal intensity both on T1 and T2- weighted images, with suppression on fay- saturated sequences [23]. Cases with diffuse fatty infiltration can be difficult to differentiate from thyrolipomatosis. Fine-needle aspiration biopsy of the goiter is a highly useful technique to have a previously ruled out a malignant process, such as anaplastic carcinoma or lymphoma [24].

There aren’t effective treatment of systemic amyloidosis available. Colchicine may prevent amyloid deposition in familial Mediterranean fever [25]. The treatment of choice in patients with amyloid goiter is total thyroidectomy to solve compression symptoms. The final diagnosis is based on histological examination of the surgical specimen.

## Conflicts of interest

No conflicts of interest.

## Sources of funding

No funding received.

## Ethical approval

Ethical approval by Department of Medical and Surgical Sciences and Advanced Technologies, G.F. Ingrassia.

## Consent

Informed consent was received from the patient.

## Author contribution

Cannizzaro MA: Study design, revision.

Lo Bianco S: Write Study, revision.

Saliba W: write study, revision.

D’Errico S: write study.

Pennetti Pennella F: write study.

Buttafuoco G: write study.

Provenzano D: Revision.

Magro G: write study, study design.

## Registration of research studies

Not needed.

## Guarantor

Cannizzaro Matteo Angelo.

## Provenance and peer review

Not commissioned, externally peer reviewed.
